# Citrate Functionalized Zirconium-Based Metal Organic Framework for the Fluorescent Detection of Ciprofloxacin in Aqueous Media

**DOI:** 10.3390/mi13122097

**Published:** 2022-11-28

**Authors:** Bo-Tau Liu, Dillirani Nagarajan, Selvam Kaliyamoorthy, Balamurugan Rathinam

**Affiliations:** 1 Department of Chemical and Materials, Engineering National Yunlin University of Science and Technology, Yunlin 64002, Taiwan; 2 Department of Chemical Engineering, National Cheng Kung University, Tainan 70101, Taiwan; 3 The Noyori Laboratory, Graduate School of Science and Research Center for Materials Science Nagoya University, Furo-Cho, Chikusa-ku, Nagoya 464-8602, Japan

**Keywords:** MOF-808, zirconium, ciprofloxacin, fluorescent detection, antibiotics

## Abstract

Ciprofloxacin (CIP) is a commonly used antibiotic for the treatment of infectious diseases in humans and as a prophylactic agent in the livestock industry, leading to the environmental discharge of significant amounts of CIP. CIP is stable in aquatic systems leading to its pseudo-persistence. Constant exposure to these antibiotics results in the generation of antibiotic-resistant pathogens and potential toxicity/hypersensitivity in humans. Therefore, it is necessary to develop a convenient, rapid, and cost-effective method for the monitoring of ciprofloxacin in environmental samples. Rhodamine-based fluorescent receptors have the limitation of aqueous solubility. Therefore, in order to overcome this drawback, we designed a novel fluorescent receptor based on a zirconium-based metal organic framework (MOF-808). The precursor, MOF-808, was synthesized and functionalized by using sodium citrate to obtain a receptor called C-MOF-808. The C-MOF-808 was structurally characterized by XRD and spectroscopic analyses. Thus, this synthesized receptor can be used for the fluorescent detection of CIP in aqueous media with a detection limit of 9.4 µM. The detection phenomena of the receptor were studied by absorption as well as fluorescent spectra. The binding behavior of CIP with the receptor was studied by FT-IR and ^1^H-NMR analyses, and a binding mechanism is proposed.

## 1. Introduction

Metal–organic frameworks (MOFs) are class of coordination polymers built from organic–inorganic hybrid materials, in which organic ligands and metal ion/clusters act as struts and nodes, respectively [1,2,3,4,5,6]. Owing to their unique features of diverse compositions, adjustable porosity/tunable pore size, large surface area, facile synthesis, enhanced catalytic activity, easy surface functionalization, and biocompatibility, MOFs have gained significantly increasing attention especially for chemical and bio-sensing applications [7]. Among the MOFs, zirconium-based MOFs are increasing in attention as the consequence of their exceptional chemical, hydrothermal, and mechanical stabilities [8], which alleviate the limitations to their uses in industrial processes [9,10,11,12,13,14,15]. For example, a zirconium-based MOF called MOF-808, possessing the structure of [Zr_6_O_4_(µ3-OH)_4_(OH)_6_(BTC)_2_]. nH_2_O exhibits excellent catalytic activity under both acidic and neutral pH reaction conditions, which has increased its use in biological applications [8,16]. Due to its peroxidase-like activity at neutral pH, MOF-808can be used for colorimetric biosensing, for example, the sensing of hydrogen peroxide (H_2_O_2_) when used as an oxidant for 3,3’,5,5’-tretramethylbenzidine [8] and fluorescent sensors [17,18,19,20,21]. 

For the treatment of respiratory and digestive infections in humans and in livestock excrement, soil, and even water, a fluoroquinolone antibiotic called ciprofloxacin (CIP), with long residence times due to the unparalleled stability and fabulous germ-proof effects, is extensively used [22,23,24,25]. However, this antibiotic also exhibits adverse effects, especially inducing and engendering poisonousness in aquatic organisms [25]. This is also expressed in the meat and milk of animals that are treated with CIP, which leads to potential toxicity [26]. Therefore, there is an urgent need to develop a novel method for the detection/monitoring of CIP in samples. Due to expensive/sophisticated instrumentation, requirement of laborious sample-preparation steps, rigorous experimental conditions, and highly skilled operational for the available electrochemical [27,28] and liquid chromatography techniques [29,30], the finding of an alternative method is highly demanded. Fluorescent methods offer the advantages of simplicity, a rapid response, sensitivity, a low cost, a non-destructive methodology, real-time monitoring, and a high spatial resolution via microscopic imaging, which can overcome the abovementioned limitations [22,31,32,33,34,35,36,37,38,39,40,41,42]. Hence, fluorophore based on an organic molecule such as rhodamine dye has the drawback of aqueous solubility, which lime the biological applications. Therefore, in this work, we designed a citrate-functionalized zirconium-based metal organic framework (MOF-808) as a receptor for the fluorescent detection of ciprofloxacin (CIP) in aqueous media at a physiological pH. The receptor exhibited high fluorescence in the presence of CIP, and its detection behavior was studied by using absorption and emission profiles, and the results are discussed in detail. 

## 2. Materials, Methods, and Experiments

Zirconyl chloride octahydrate (ZrOCl_2_.8H_2_O), benzene tricarboxylic acid (H_3_BTC), and all the other chemicals were purchased from Aldrich Chemicals and used without further purification. All the solvents including DMF and formic acid used for the syntheses were spectroscopic grade and were used as received. Fourier-transform infrared spectra (FTIR) was recorded on a Jasco VALOR III Fourier-transform infrared spectrophotometer. Nuclear magnetic resonance (NMR) spectra were obtained on a Bruker AMX-500 high-resolution NMR spectrometer in deuterated solvents. UV/Vis absorption spectra were measured with a Jasco V-550 spectrophotometer. The fluorescence spectra were measured with a Hitachi F-4500 fluorescence spectrophotometer at λ_ex_ = 310 nm. To measure the optical responses of the receptor (C-MOF-808), 1 micromolar solution of the receptor was titrated against CIP. 

### 2.1. Synthesis of MOF-808

**Figure 1:**
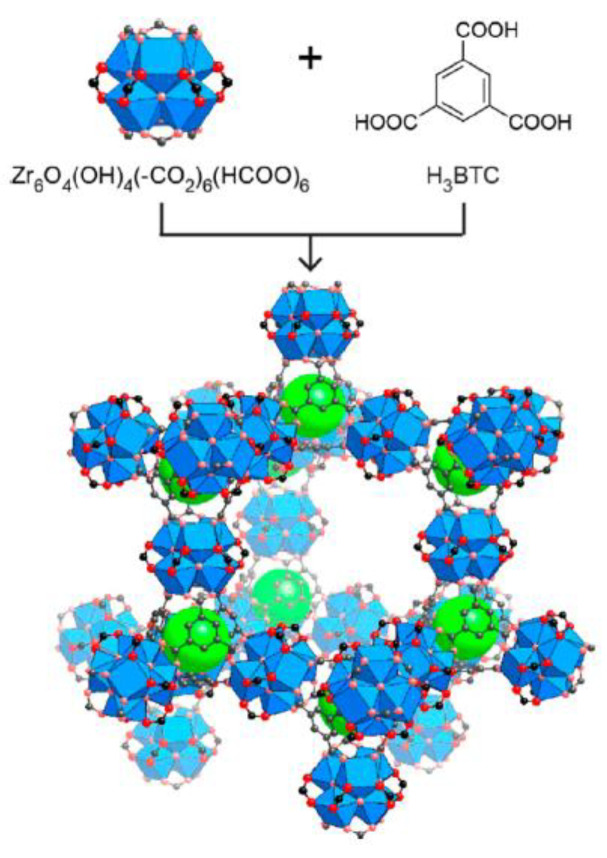
Synthesis of MOF-808 (structure is reprinted with permission from *J. Am. Chem. Soc.* 2014, 136, 37, 12844–12847; copyright 2022 American Chemical Society) [12].

The synthesis of MOF-808 and the receptor C-MOF-808 were synthesized according to the reported procedures in the literature [12]. The typical synthesis of MOF-808 is as follows: About 2.58 g of ZrOCl_2_.8H_2_O and 0.84 g of H_3_BTC were taken in a 500 mL glass flask and dissolved in the mixture of DMF/Formic acid (100 mL/100 mL) by ultrasonic in a 40 KHZ ultrasonic cleaner. The obtained solution was continuously stirred and heated at 150 °C for 6 h, and then allowed to cool to room temperature. The solution was subjected to centrifugation and washed three times with absolute alcohol, followed by drying in an oven at 60 °C for 24 h, which resulted in MOF-808 as white powder (Figure 1). 

### 2.2. Synthesis of Receptor (C-MOF-808)

**Figure 2:**
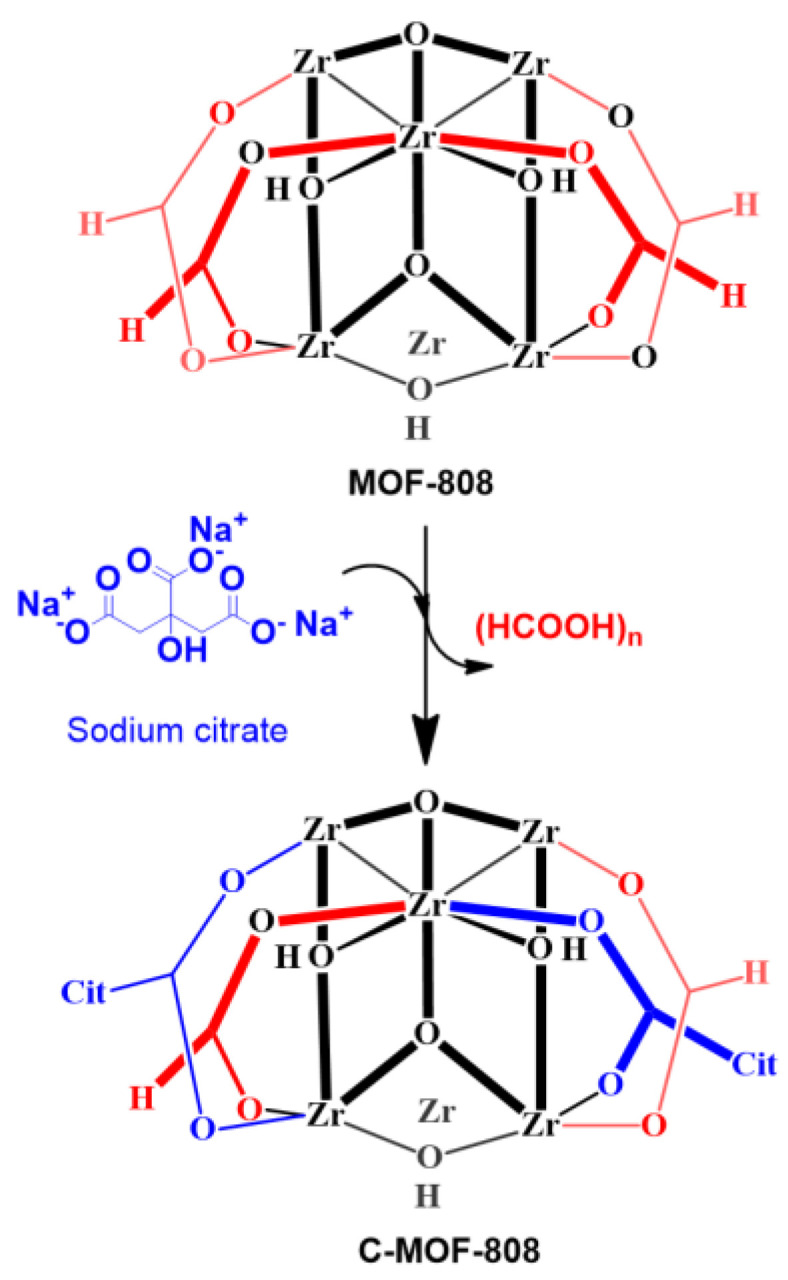
Synthesis of receptor C-MOF-808.

The receptor called C-MOF-808 was obtained by functionalization of MOF-808 as follows. About 1 g of the above-synthesized MOF-808 was added to 20 mL of water, and then 3 g of sodium citrate was added to the solution; the resulting mixture was allowed to stir for 30 min at room temperature and then heated at 85 °C for 4 h. The milky solution become homogeneous transparent liquid, indicating the formation of C-MOF-808. Then, it was allowed to cool down to room temperature. The resulting solution was lyophilized/freeze-dried to obtain C-MOF-808 as white powder (Figure 2). 

## 3. Results and Discussion

### 3.1. Characterization of the Synthesized MOF and C-MOF-808

**Figure 3:**
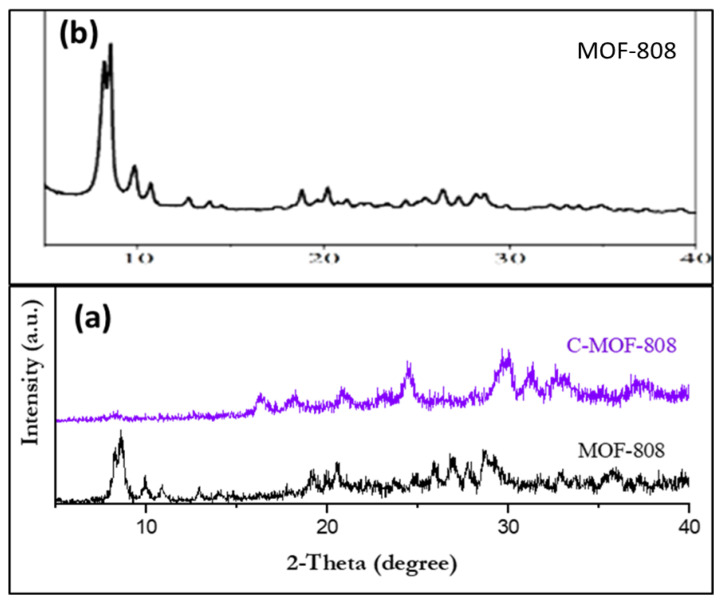
XRD patterns of (**a**) MOF and C-MOF-808; (**b**) reported XRD pattern of MOF-808 for comparison [46].

The structure of the synthesized precursor and receptor are shown in Figure 1 and Figure 2, respectively. The receptor was synthesized starting from zirconyl chloride octahydrate (ZrOCl_2_.8H_2_O), which is reacted with benzene tricarboxylic acid in the presence of formic acid and DMF. Thus, the obtained MOF-808 was further functionalized with trisodium citrate to form C-MOF-808. The successful formation of C-MOF-808 was characterized by XRD and ^1^H-NMR spectroscopy analysis. The XRD analysis of both MOF and C-MOF-808 are shown in Figure 3. It can be seen that the prepared MOFs-808 displayed peaks at 2θ = 8.6° and 9.9°, which were assigned to diffraction from the planes (**311**) and (**222**) of MOF-808, respectively, and as are in accordance with reports in the literature, indicating the successful synthesis of MOF-808 [43,44,45]. From Figure 3, it can be observed that the peak corresponding to MOF-808 was found to be slightly affected after functionalization with sodium citrate, indicating the successful functionalization of MOF-808. Moreover, after functionalization with sodium citrate, the resulting receptor, C-MOF-808, is found to be soluble in water. However, MOF-808 is not soluble in water. This also further confirmed the formation of C-MOF-808.

**Figure 4:**
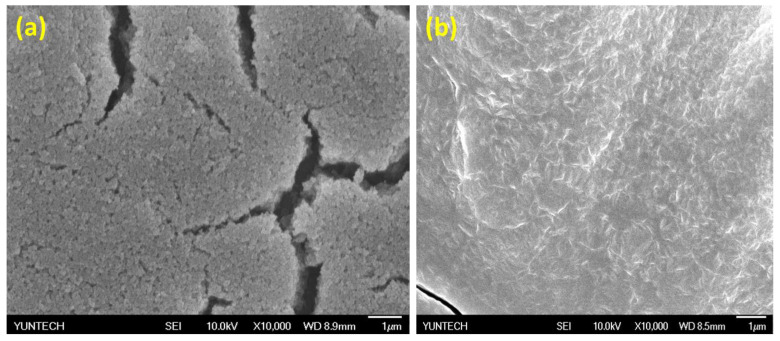
The SEM images of (a) MOF-808 and (**b**) C-MOF-808.

In addition, both MOF-808 and C-MOF-808 were subjected to morphological analysis by SEM, and the results are merged in Figure 4. As seen in Figure 4, MOF-808 exhibited nanoparticles’ morphology with the size of 50–100 nm, which is similar to the reported morphology in the literature [10]. However, C-MOF-808 exhibited a highly crystalline morphology, indicating the successful functionalization of MOF-808 by citrate. 

**Figure 5:**
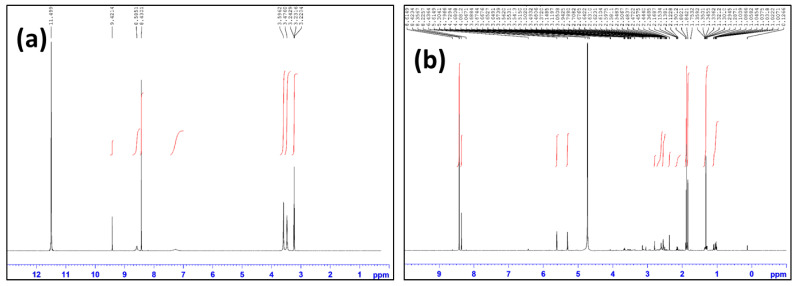
^1^H-NMR spectrum of (**a**) MOF-808 (deutriated TFA) and (**b**) C-MOF-808 (D_2_O).

Moreover, the ^1^H-NMR spectra of both MOF and C-MOF-808 were carried out, and the results are given in Figure 5. From the proton NMR of MOF-808, the presence of peaks at 8. 43 and 8.58 ppm was observed, corresponding to the aromatic protons and formate, respectively. All the peaks are in accordance with the reported references [47]. Peaks around 3 ppm are reported as the peak for the DMF solvent used during synthesis [47]. After modification by sodium citrate, the spectra showed new peaks at 1.3 and 1.8 ppm, corresponding to the -CH_3_ and –CH_2_ groups of citric acid, indicating the successful functionalization of MOF-808.

### 3.2. Detection of Ciprofloxacin (CIP) by C-MOF-808

**Figure 6:**
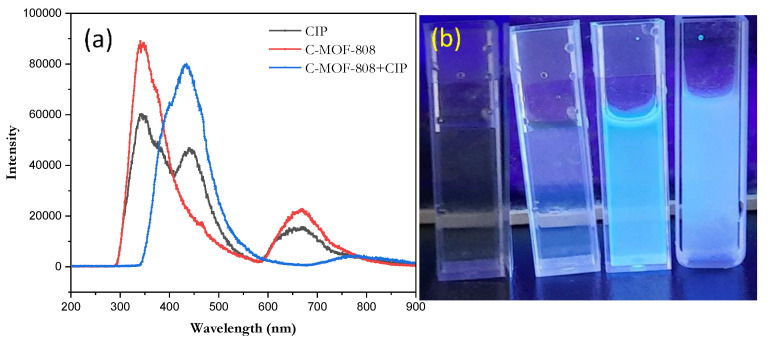
(**a**) Fluorescence spectra of receptor, CIP, and their mixture (λ_ex_ = 310 nm) and (**b**) real images of the aqueous solution of receptor with increasing concentration of CIP under UV-light.

The detection of ciprofloxacin was carried out by using the absorption and fluorescent spectroscopy methods. Aqueous solution of both the receptor (1 µM) and CIP (1 mM) was prepared. From the real images, it was observed that the aqueous solution of the receptor was colorless and had little or no fluorescence (Figure 6b). However, after the addition of CIP into the receptor, an obvious fluoresce was observed, and the fluorescent intensity was increased by increasing the concentration of CIP. The fluorescent spectral changes of the receptor with CIP are shown in Figure 6a. From the spectra, it was observed that the receptor exhibits a fluorescence signal around 350 nm and 660 nm, whereas CIP showed fluorescence peak around 350, 450, and 650 nm. Once CIP was added into the receptor, which enhanced the intensity of the fluorescence (Figure 6b), a bathochromic shift was observed (Figure 6a). This shift may be due to the addition of CIP, which affects or reduces the energy gap between the HOMO (highly occupied molecular orbital) and LUMO (lowest unoccupied molecular orbital) [48]. 

**Figure 7:**
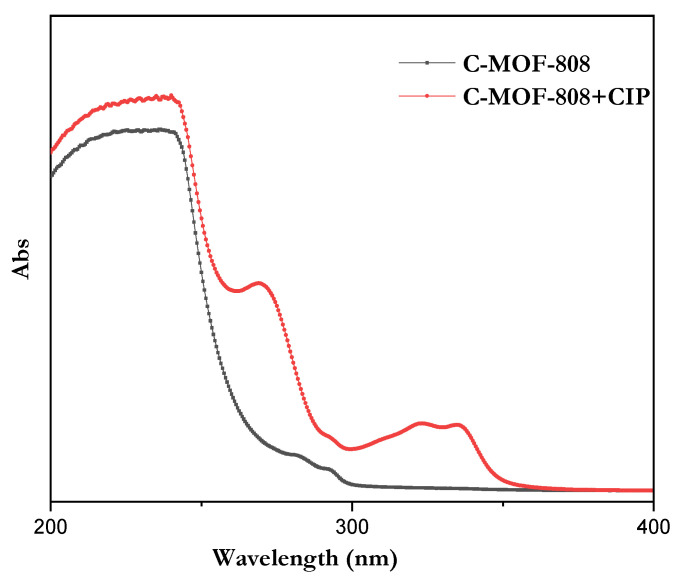
The absorption spectra of receptor and with CIP.

From the absorption spectra, there was no obvious absorption peak observed for a receptor within the range of 300 to 400 nm. However, after the addition of CIP, the absorption intensity was found to be increased at 323 and 335 nm, as shown in Figure 7.

### 3.3. Absorption and Emission–Kinetic Studies

**Figure 8:**
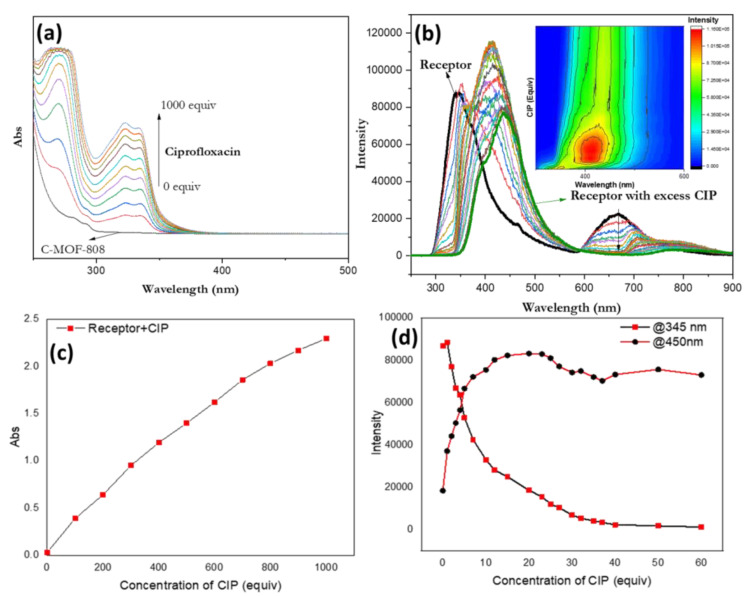
The changes in (**a**) the absorption and (**b**) emission spectra of the receptor at pH 6.9 upon titration with CIP (λ_ex_ = 310 nm; inset: contour plot). The absorbance (**c**) and emission (**d**) intensities of the receptor as a function of the equivalents of CIP added.

Therefore, absorption and fluorescence titrations were carried out for the binding of the receptor with CIP. The titrations revealed that the intensity of the absorption (Figure 8a) and emission (Figure 8b) increased at regular intervals as CIP was progressively added (up to 1000 equivalents), resulting in a brilliant blue fluorescence under UV light. An emission band centered at 345 nm in the fluorescence spectra of the receptor red shifted linearly when increasing the concentration of CIP (Figure 8b). The kinetics study demonstrated that the absorption changes were proportional to the CIP concentration (Figure 8c). In the case of the emission spectra, ratiometric detection was observed. The emission peak at 345 nm is found to be lowered (Figure 8d) and the peak at 450 nm is found to be enhanced, when increasing the concentration of CIP up to 30 equivalents, which then starts to decrease when further increasing the concentration of CIP, indicating that CIP undergoes an aggregation-induced quenching phenomena that occurs at a higher concentration. 

The detection limit (DL) can be determined from the following equation [49]: DL = K (Sb1/S); where K = 3, Sb1 is the standard deviation of the blank solution (only receptor), and S denotes the slope of the calibration curve (fluorescent spectra). In our experiments, Sb1 is calculated to 18,310 and the slope was determined to be 5835. As a result, the DL for the detection of CIP reaches to 9.4 µM, which is comparable with that reported in the literature [22]. Most of the reported sensors for the fluorescent detection of CIPs are lanthanides (La/Eu/Tb)-based coordination polymer nanoparticles, and their detection limits are varied, in the range of 0.0136 µM–780 nM [39,41,42]. Our designed receptor consists of MOF nanoparticles without doping of the lanthanides; however, it exhibited the detection limit in micromolar concentrations. Moreover, our receptor is simple, cost-effective, and easy to synthesize with an enhanced aqueous solubility over the other reported receptors.

### 3.4. Binding Mechanism of the Receptors towards CIP

**Figure 9:**
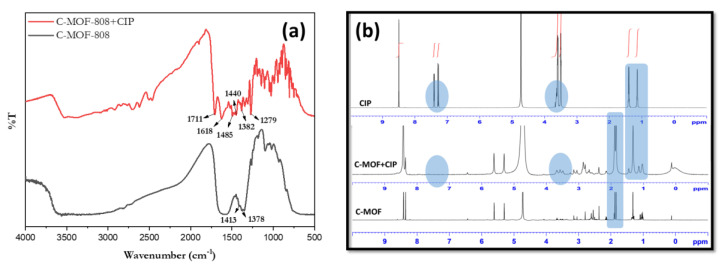
(**a**) FTIR spectra of the receptor (C-MOF-808) and the analyte (CIP) and (**b**) ^1^H-NMR spectra of receptor, CIP, and receptor with CIP in D_2_O.

To understand the binding mechanism of the receptor with CIP, it was subjected to FTIR, and the results are merged in Figure 9a. From the spectrum of the receptor (C-MOF-808), the broad peak around 1650–1550 cm^−1^ corresponds to the –COO stretching mode of the receptor. Moreover, the bands in the 1413 cm^−1^ and 1378 cm^−1^ regions are related to the C = C bond and the stretching mode of O–H in the aromatic compound of the receptor, suggesting the formation of C-MOF-808. From the spectra of the receptor with CIP, it can be observed that the crystalline CIP had many characteristic peaks at the fingerprint (1200–1800 cm^−1^) [50]. There was a peak that appeared in 1711 cm^−1^ due to the fact that the vibration of the carboxylic acid (C=O) and the vibration of the ketone (C=O) were located at 1618 cm^−1^ [51,52]. In addition, two relatively strong peaks appeared at 1279 cm^−1^ and 1485 cm^−1^, corresponding to the O–H deformation vibration and C-O vibration of CIP, indicating the interaction of CIP with the receptor. The characteristic peaks of the receptor such as 1413 cm^−1^ and 1378 cm^−1^ were found to be slightly shifted to 1440 cm^−1^ and 1382 cm^−1^, which further confirmed the interaction of CIP with the receptor. Moreover, these compounds were subjected to ^1^H-NMR spectral analysis, and their results are merged in Figure 9b.

As seen in Figure 9b, the presence of a peak at 1.8 ppm for C-MOF-808, which corresponds to the methyl group, indicates the successful functionalization of MOF-808 with acetic acid (C-MOF-808). The abovementioned peaks were also observed in C-MOF-808+CIP, indicating the presence of C-MOF-808 in the mixture. The peaks at around 8.3–8.4 ppm correspond to the aromatic protons of C-MOF-808. The peaks at 1.15, 1.35, and 3.65 ppm for the CIP corresponding to the –CH_2_ and -CH groups of the cyclopropyl unit were found to be shifted to 1.3 and 1.4 (only for the CH_2_ protons) in the mixture, indicating the interaction of CIP with C-MOF-808. The peak at 4.8 ppm corresponds to the solvent peak of D_2_O. Moreover, the peaks at 7.4 and 7.5 ppm for CIP corresponding to the aromatic protons of the CIP were found to be shifted and merged with the aromatic protons of C-MOF-808 around 8.5-8.7 ppm in the mixture, indicating the interaction of CIP with the receptor.

**Figure 10:**
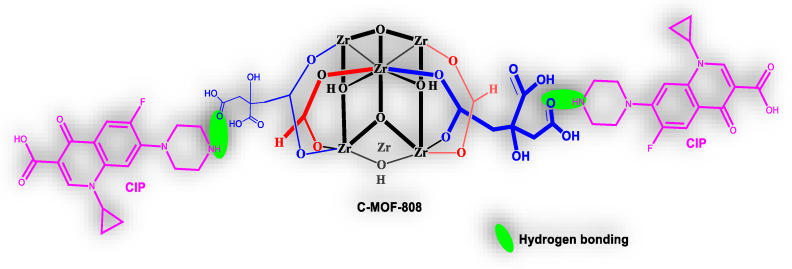
The schematic representation of the proposed mechanism of binding of CIP with the receptor.

Based on this evidence, it was concluded that the CIP can interact with the receptor. The receptor structurally possessing the carboxylic acid group (-COOH) and CIP has a piperazine –NH group. Therefore, there is the possibility of H-bonding between the –NH group of the CIP and the –COOH group of the receptor, which altered the absorption and fluorescent properties of the receptor. This strongly suggests that the fluorescent enhancement by CIP is due to the H-bonding induced emission (HBIE) in water, as reported by Haifang Liu and coworkers [53]. Based on the structures of the receptor and analyte, the schematic representation of the proposed mechanism for the possible binding of CIP with the receptor is given in Figure 10.

## 4. Findings and Discussion

Generally, in fluorescent sensors, the non-aqueous solubility of the receptor is the main drawback that limits their utilization in both environmental and biological applications. In order to overcome the abovementioned limitations, the development of water-soluble fluorescent nanomaterials has received great attention in recent years. Water-soluble fluorescent nanomaterials can be achieved by proper functionalization of the nanomaterials. Here, we achieved aqueous solubility by the functionalization of MOF-808 using citrate, and, thus, the developed receptor can detect CIP in aqueous media with the detection limit of micromolar solutions. Very interestingly, the CIP-induced sensitized emission band is fully separated from both receptor and analyte, which would enable us to detect Cipro by using a common fluorimeter. Hydrogen bonding induced emission (HBIE) is the cause of the changes in the emission spectra when CIP was added. Hence, improving the detection limit to the picomolar level is a still challenging task, one which is in progress.

## 5. Conclusions

In conclusion, we have designed and synthesized a novel fluorescent receptor based on a citrate-functionalized metal–organic framework consisting of zirconium metal ions (C-MOF-808), which was characterized by XRD and ^1^H-NMR spectroscopy analysis. Our synthesized receptor is colorless and less fluorescent. It shows high sensitivity towards ciprofloxacin (CIP). The sensing behavior was studied by using absorption and fluorescent spectroscopy. Increasing the concentration of CIP increased the absorption intensity and the red-shifting of the fluorescent intensity when compared to that of neat CIP or C-MOF-808, indicating the detection of CIP. The receptor, before and after binding with CIP, was studied by using FTIR and ^1^H-NMR spectra and compared with neat CIP. The results showed that CIP has a strong interaction/binding with C-MOF-808. Based on the structures of C-MOF-808 and CIP, a possible detection mechanism was proposed. The hydrogen bonding between the carboxylic group (-COOH) of the C-MOF-808 with the –NH group of the CIP may alter the absorption as well as the fluorescent profiles of the receptor, indicating the phenomena of hydrogen bonding induced emission (HBIE). Due to the aqueous solubility of C-MOF-808, it can be utilized for rapid and in situ imaging of the nucleus in living cells in a fluorescence turn-on mode, which has a great practicability to be used for the nucleus imaging in bioanalytical studies, especially detection of CIP in urinary samples and clinical applications.

## Data Availability

Not applicable.
